# Comprehensive characterization of the tumor microenvironment for assessing immunotherapy outcome in patients with head and neck squamous cell carcinoma

**DOI:** 10.18632/aging.103460

**Published:** 2020-11-18

**Authors:** Jian Zhang, Xi Zhong, Huali Jiang, Hualong Jiang, Tao Xie, Yunhong Tian, Rong Li, Baiyao Wang, Jiexia Zhang, Yawei Yuan

**Affiliations:** 1 Department of Radiation Oncology, Affiliated Cancer Hospital & Institute of Guangzhou Medical University, State Key Laboratory of Respiratory Diseases, Guangzhou Institute of Respiratory Disease, Guangzhou, 510095, P. R. China; 2 Department of Radiation, Affiliated Cancer Hospital & Institute of Guangzhou Medical University, Guangzhou, 510095, P. R. China; 3 Department of Cardiovascularology, Tungwah Hospital of Sun Yat-sen University, Dongguan, 523000, P. R. China; 4 Department of Urology, Tungwah Hospital of Sun Yat-sen University, Dongguan, 523000, P. R. China; 5 State Key Laboratory of Respiratory Disease, National Clinical Research Center for Respiratory Disease, Guangzhou Institute of Respiratory Health, the First Affiliated Hospital of Guangzhou Medical University, Guangzhou, 510120, P. R. China

**Keywords:** head and neck squamous cell carcinoma, tumor microenvironment, immunotherapy, single nucleotide variants, copy number variations

## Abstract

The tumor microenvironment (TME) constitutes a complex milieu of cells and cytokines that maintain equilibrium between tumor progression and prognosis. However, comprehensive analysis of the TME and its clinical significance in head and neck squamous cell carcinoma (HNSCC) remains to be unreported. In this study, based on large-scale RNA sequencing data pertaining to single nucleotide variants (SNVs) and copy number variations (CNVs) in HNSCC patients from The Cancer Genome Atlas database, we analysed subpopulations of infiltrating immune cells and evaluated the role of TME infiltration pattern (TME score) in assessing immunotherapy outcome. TME signature genes involved in several inflammation and immunity signalling pathways were observed in the TME score subtype, which were considered immunosuppressive and potentially responsible for significantly worse prognosis. In comparison with SNV- and CNV-mediated tumor mutation burden, TME score can significantly differentiate between high- and low-risk HNSCC and predict immunotherapy outcome. Our data provide clarity on the comprehensive landscape of interactions between clinical characteristics of HNSCC and tumor-infiltrating immune cells. TME score seems to be a useful biomarker that can predict immunotherapy outcome in HNSCC patients.

## INTRODUCTION

Head and neck squamous cell carcinoma (HNSCC), one of most common cancers in the world, is an aggressive and frequently lethal head and neck malignancy [1]. Despite advances in diagnostic and therapeutic approaches, the 5-year survival rate of patients with HNSCC is only approximately 50%–60% [2]. HNSCC is associated with distinct clinical and biological heterogeneity; patients with aggressive disease are managed using cetuximab, an anti-EGFR antibody, but only around 13% metastatic patients respond to such treatment [3, 4]. Phase III clinical trials of programmed death (PD)-1 immune checkpoint inhibitors (ICIs) (pembrolizumab and nivolumab) and PD-ligand (L)1 (durvalumab and avelumab) ICI immunotherapy have been performed in patients with recurrent and metastatic HNSCC. Pembrolizumab combined with platinum and fluorouracil was found to outperform the cetuximab-based platinum and fluorouracil combination in terms of overall survival (median, 13.6 vs. 10.1 months) when administered as the first-line of treatment for recurrent and metastatic HNSCC. Nevertheless, nearly 64% patients still did not respond to the PD-1/PD-L1 ICI immunotherapy, indicating innate, adapted, or quickly acquired resistance to the treatment [5–8]. Unfortunately, little is known about mechanisms underlying the response of patients with HNSCC to immunotherapy.

The tumor microenvironment (TME) is composed of transformed cells, infiltrating immune cells and stromal cellular elements. Tumor-infiltrating immune cells are known to substantially influence therapeutic responses and clinical outcomes [9]. For instance, tumor-associated macrophages and regulatory T cells are associated with pro-tumor functions [10–12], whereas tumor-infiltrating lymphocytes and CD8+ T effector cells have been associated with improved clinical outcomes and better response to immunotherapy [13–15]. However, the mechanisms by which immune infiltration affects immunotherapy outcome in patients with HNSCC remain poorly understood.

With the application of high-throughput sequencing, a comprehensive landscape of genomic and transcriptomic alterations in HNSCC has emerged from The Cancer Genome Atlas (TCGA) database, permitting the analysis of, for instance, DNA methylation, single nucleotide variants (SNVs) and copy number variations (CNVs) [16, 17]. Several recurrent chromosomal region copy number alterations have been found to affect broad and focal chromosomal regions, leading to the activation of multiple candidate driver genes [18]. Alterations in associated pathways, such as p53, mitogen-activated protein kinase, phosphatidylinositol 3′–kinase-Akt and nuclear factor kappa-B signalling pathways, may elicit complementary or synergistic effects that are important in tumorigenesis [19]. However, whether genomic alterations drive corresponding changes in the TME in HNSCC remains to be comprehensively elucidated; moreover, their functional interactions and roles in immunotherapy outcome and clinical prognosis require further exploration.

Herein based on large-scale RNA sequencing (RNA-seq) data pertaining to patients with HNSCC from TCGA database, we investigated 22 subpopulations of infiltrating immune cells using the CIBERSORT algorithm. We analysed TME infiltration pattern (TME score) and systematically correlated TME phenotypes with genomic characteristics and clinicopathological features of HNSCC. We found TME score to be an effective prognostic biomarker and it seems useful for predicting immunotherapy outcome in patients with HNSCC.

## RESULTS

### Overview of TME characteristics in HNSCC

**Figure 1:**
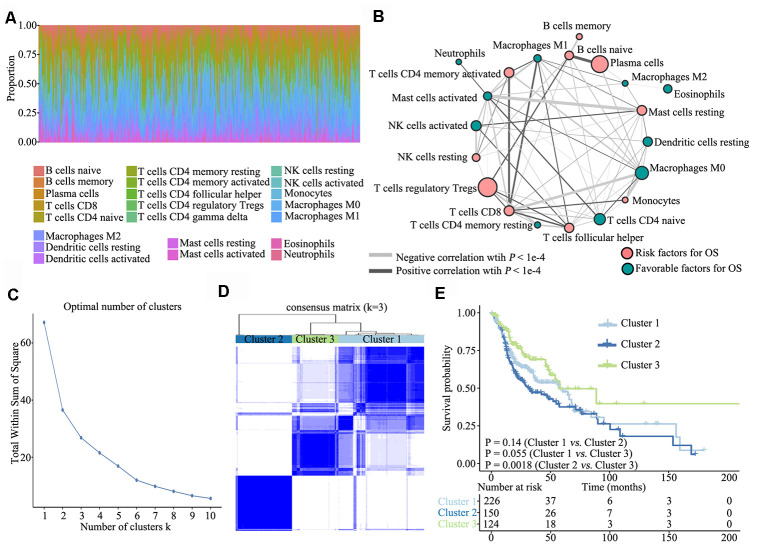
**Overview of TME characteristics.** (**A**) Relative percentage of each immune cell type in 502 patients with HNSCC from TCGA database. (**B**) Tumor–immune cell interactions. The size of each cell represents the impact of each TME cell type on survival and was calculated using log10 (log-rank test P value). Risk factors for overall survival are indicated in pink, and favourable factors are in green. The lines connecting TME cell types represent cellular interactions. The thickness of the lines represents the strength of correlation, which was estimated using Spearman correlation analysis. Negative correlation is indicated in grey and positive correlation in black. (**C**) The elbow criterion determines the optimal number of TME clusters (K = 3). (**D**) Consensus clustering analysis identification of the three TME clusters (samples, n = 500). The white (consensus value = 0, samples never clustered together) and blue (consensus value = 1, samples always clustered together) heatmap display sample consensus. (**E**) Kaplan–Meier curves for survival probability of the three clusters. Log-rank test was used for data analysis.

To investigate TME characteristics in HNSCC, based on the RNA-seq data of 502 patients with HNSCC from TCGA database, we systematically investigated 22 subpopulations of infiltrating immune cells using CIBERSORT. The distribution ratio of infiltrating immune cells among the samples showed a significant difference (Figure 1A), and the TME cell network depicted a comprehensive landscape of tumor–immune cell interactions, cell lineages and their effects on the overall survival (OS) of patients with HNSCC (Figure 1B; Supplementary Table 1).

Further, we performed unsupervised consensus clustering of all tumor samples for the molecular classification of HNSCC. The optimal number of clusters was determined by the K value. After assessing relative changes in the area under the cumulative distribution function curve and consensus matrix heatmap, we selected a three-cluster solution (K = 3), which showed no appreciable increase in the area under the cumulative distribution function curve (Figure 1C). To gain further insights into the molecular heterogeneity of HNSCC, unsupervised consensus clustering was performed to explore discernible patterns of the TME clusters. Based on the consensus matrix heatmap, three distinct TME-based molecular clusters were identified (Figure 1D). To further clarify the clinical implications of these clusters, we performed Kaplan–Meier curve analyses to elucidate the association between them and clinical prognosis. Notably, survival analysis based on the TME phenotypes indicated that TME cluster 3 (n = 124) was significantly associated with better prognosis and that TME cluster 2 (n = 150) was associated with poorer prognosis (log-rank test, P = 0.0018). Of the 500 patients with HNSCC, 226 belonged to TME cluster 1, which was associated with intermediate prognosis (log-rank test, P > 0.05) (Figure 1E).

### Signature and functional annotation of the TME clusters

**Figure 2:**
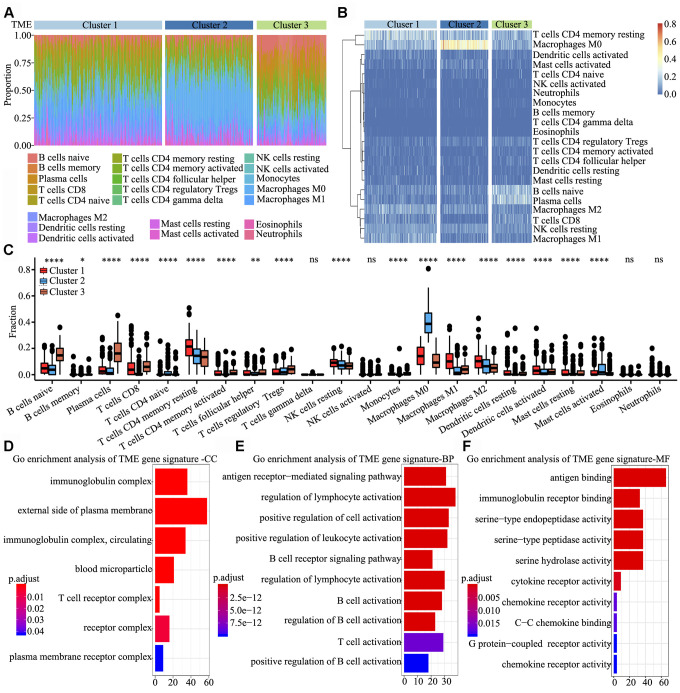
**Signature and functional annotation of the TME clusters.** (**A**) Relative percentage of each immune cell type in the three TME clusters. (**B**) Unsupervised hierarchical clustering of the clusters. (**C**) Relative populations of TME cells present in the three clusters. Within each group, the scattered dots represent expression values of TME cells. We also plotted the Immunoscore for the three clusters. The thick line represents the median value. The lower and upper ends of the boxes are the 25^th^ and 75^th^ percentiles. The whiskers encompass 1.5 times the interquartile range. Statistical differences in the three clusters were compared using the Kruskal–Wallis test. The range of P values are labelled above each boxplot with asterisks (* P < 0.05, ** P < 0.01, *** P < 0.001, ****P < 0.0001, ns = not significant). (**D**–**F**) Gene ontology enrichment analysis of TME signature genes in the cellular component (**D**), biological process (**E**) and molecular function (**F**) categories. The x-axis indicates the number of genes within each gene ontology term.

To determine the characteristics of immune cells in the three TME clusters, infiltrating immune cell subpopulations were analysed using CIBERSORT. Tumor ploidy and malignant cell purity showed no differences among the three clusters (Supplementary Figure 1A and 1B), whereas the proportion of infiltrating immune cells among the clusters showed significant differences, particularly that of resting memory CD4+ T cells, M0 macrophages, naïve B cells and plasma cells (Figure 2A). Further, the clusters were subjected to unsupervised hierarchical clustering, and the obtained results revealed that the 22 subpopulations of infiltrating immune cells showed differential patterns among the clusters (Figure 2B). To identify and elucidate differences in infiltrating immune cells, the relative fraction of 22 leukocyte subpopulations in each sample was estimated based on the differential expression of 547 genes [20]. Comparing the TME clusters revealed several differences, including increased levels of naïve B cells, regulatory T cells and eosinophils; in contrast, the levels of resting memory CD4+ T cells, resting NK cells and M2 macrophages were markedly reduced (Figure 2C).

To identify biological characteristics underlying each TME phenotype, differentially expressed genes (DEGs) among the three clusters were analyzed using the limma package. In total, 306, 998 and 676 DEGs were obtained upon three comparisons (TME cluster 1 vs. TME cluster 2, TME cluster 2 vs. TME cluster 3 and TME cluster 1 vs. TME cluster 3). Overall, 622 common DEGs were further screened in the comparisons. Next, we used random forest algorithms for dimension reduction to extract phenotype signatures. Unsupervised hierarchical clustering was based on the expression of the 312 most representative D EGs (Supplementary Table 2). Gene ontology enrichment analysis of TME signature genes was conducted using the R package. Significantly enriched biological processes are summarized in Supplementary Table 3. Analyses of TME signature mRNAs indicated that within the cellular component category, immunoglobulin complex, external side of plasma membrane, T cell receptor complex and receptor complex were significantly annotated (Figure 2D). Further, within the biological process category, antigen receptor-mediated signalling pathway, regulation of lymphocyte activation, positive regulation of cell activation and positive regulation of leukocyte activation were significantly annotated (Figure 2E), and within the molecular function category, pathways involved in antigen binding, immunoglobulin receptor binding and cytokine receptor activity were significantly annotated (Figure 2F). These findings indicated that the immune landscape of the three TME clusters plays a key role in immune regulation.

### Clinical characteristics of the TME phenotypes

**Figure 3:**
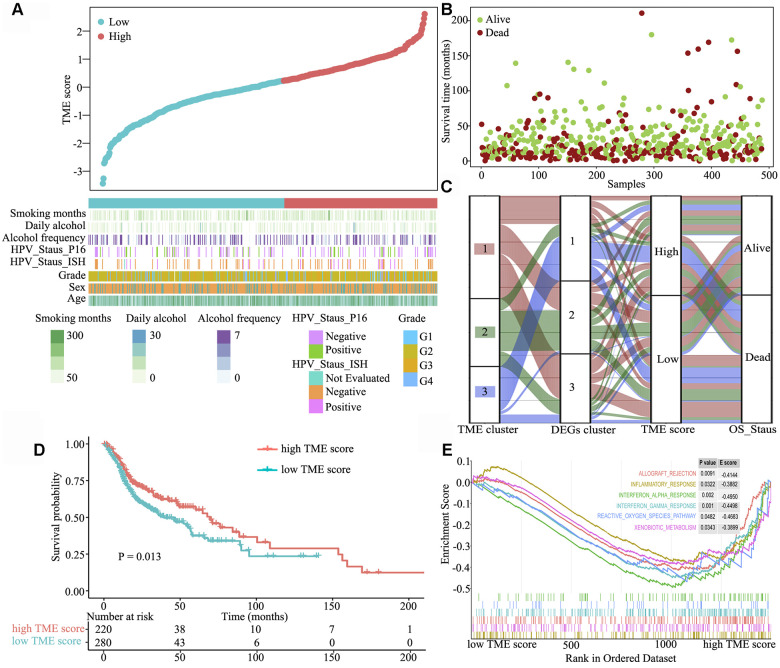
**Clinical characteristics of the TME phenotypes.** (**A**) TME score distribution in patients with HNSCC. (**B**) Overall survival status of patients with HNSCC. (**C**) Alluvial diagram of TME gene clusters in groups with different TME clusters, DEG clusters, TME scores and survival outcomes. (**D**) Gene set enrichment analysis of hallmark gene sets between the high and low TME score groups. (**E**) Kaplan–Meier curves for the high and low TME score groups. As evident, the high TME score group was associated with better outcomes than the low TME score group (log-rank test, P < 0.001).

Next, we systematically classified the TME clusters according to the gene coefficient value and calculated TME score. The maxstat R package was used to identify TME score breakpoint. The distribution of risk scores and overall survival status of patients are shown in Figure 3A and 3B, respectively. TME score was calculated for all patients, and the TME score breakpoint was used to classify them into the high (n = 220) and low (n = 280) TME score groups. High TME score and was associated with favourable outcomes, and low TME score and was associated with poor outcomes (Figure 3C). Kaplan–Meier curve and Cox regression analyses further suggested that patients in the high TME score group had significantly better OS probability than those in the low TME score group [HR 1.401 (1.074–1.827), log-rank test, P = 0.013] (Figure 3D). To understand the pathway two TME score groups involved in, gene set enrichment analysis was performed, which indicated that allograft rejection (E score = −0.414; P = 0.0091), inflammatory response (E score = −0.388; P = 0.032), interferon-α response (E score = −0.495; P = 0.002), interferon-γ response (E score = −0.450; P = 0.001), reactive oxygen species (E score = −0.468; P = 0.048) and xenobiotic metabolism (E score = −0.390; P = 0.0343) pathways were significantly downregulated in the high TME score group (Figure 3E).

### Somatic mutations of the TME phenotypes

**Figure 4:**
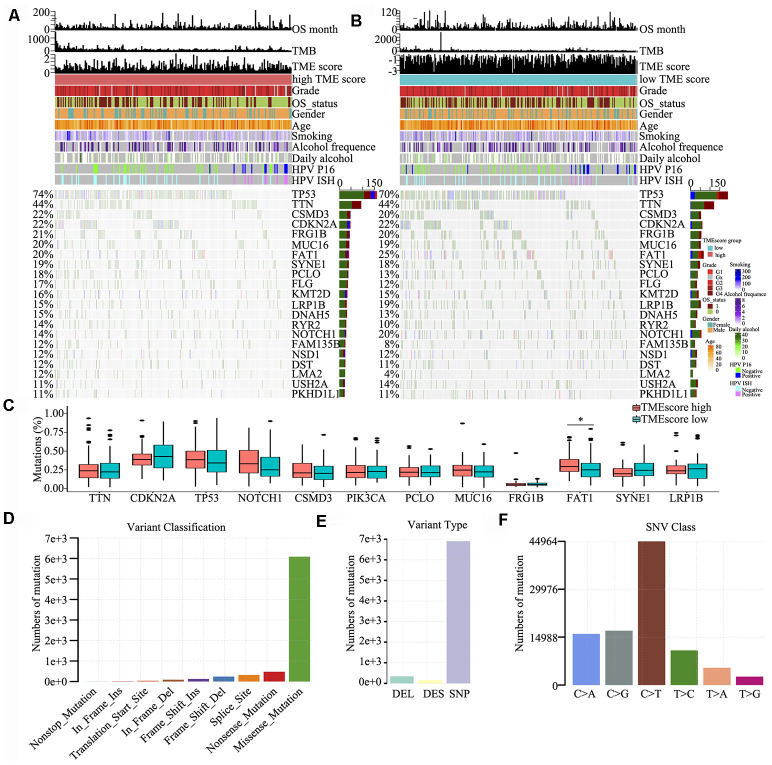
**Somatic mutations in HNSCC.** (**A**, **B**) Distribution of highly variant mutated genes correlated with TME score groups. The upper bar plot indicates overall survival (OS), TMB and TME score for each patient, whereas the left bar plot shows the mutation frequency of each gene in separate TME score groups [high (**A**) and low (**B**) TME score groups]. TME score, grade, overall survival status, gender, age, smoking, alcohol frequency, daily alcohol, HPV P16 status and HPV ISH status are shown as patient annotations. (**C**) Mutation percentage of common mutated genes in the TME score groups. (**D**) Genome variant classification. (**E**) Genome variant type. (**F**) Single nucleotide variant class.

To detect driver mutations in the TME, we analysed HNSCC-related SNP data and consequently detected 21 putative driver genes, including TP53, TTN, CSMD3, CDKN2A and NOTCH1 (Figure 4A and 4B), which were associated with the TME, using random forest algorithm with 1000 iterations. The TP53 gene showed the highest mutation rate (74% vs. 70% in the high and low TME score groups, respectively), which was consistent with the findings of other studies [21–25]. The somatic mutations of TP53, TTN, CSMD3, CDKN2A, FRG1B, FAT1 and NOTCH1 were all >20% in the high and low TME score groups; in particular, FAT1 was significantly different between high TME score and low TME score groups (P < 0.05) (Figure 4C). We also found that several genes involved in the cell cycle pathway (e.g., TP53, CDKN2A, NOTCH1 and PIK3CA) were frequently altered. Clinical reports have described associations between individual altered genes and response or resistance to ICIs [26, 27]. These mutations may be associated with a change in the TME. Consequently, we further detected and classified candidate somatic mutations, which led to the identification of nine classifications; nonstop, in-frame (insert and delete), frame-shift (insert and delete), translation start, splice, nonsense and mutations were the common somatic mutations (Figure 4D). Mutation type and spectrum analyses showed that single nucleotide mutations, particularly C > T transitions at TpCpW trinucleotide sites, were the predominant type of mutations (Figure 4E, 4F).

### Mutational signatures

**Figure 5:**
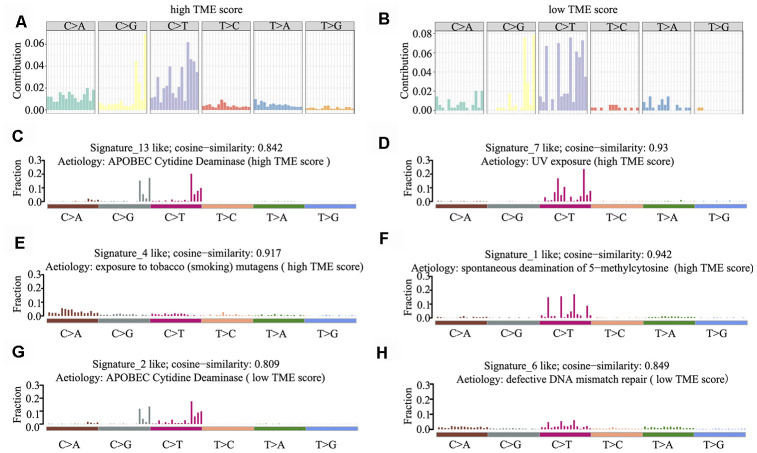
**Mutational signature of the TME score groups.** (**A**, **B**) Distribution of mutation type frequency in the high (**A**) and low (**B**) TME score groups. (**C**–**H**) Mutational signatures identified in the high (**C**–**F**) and low (**G**–**H**) TME score groups, respectively. The y-axis indicates exposure of 96 trinucleotide motifs to overall signature. The plot title indicates best match against validated COSMIC signatures and cosine similarity value along with the proposed aetiology.

Mutational signatures in the cancer genome might reflect and help trace DNA damage caused by DNA-damaging agents to which cells have been exposed. Thus, we counted the number of SNVs in the matrix of 96 possible mutations occurring in a trinucleotide context in each HNSCC sample and found that the predominant mutations were C > T and/or C > G transitions at TpCpW trinucleotide sites in both the high and low TME score groups (Figure 5A, 5B).

Using 1,000 iterations of non-negative matrix factorization [28], we then performed cosine similarity analyses to compare mutational signatures in HNSCC with current COSMIC; consequently, six independent and stable mutational signatures were identified. *De novo* signatures identified in the high TME score group were enriched in APOBEC cytidine deaminase signature (COSMIC Signature 13; cosine similarity: 0.842), UV exposure (COSMIC Signature 7; cosine similarity: 0.93), tobacco mutagens (COSMIC Signature 4; cosine similarity: 0.917) and spontaneous deamination of 5-methylcytosine (COSMIC Signature 1; cosine similarity: 0.942) (Figure 5C–5F). In contrast, *de novo* signatures identified in the low TME score group were enriched in APOBEC cytidine deaminase signature (COSMIC Signature 13; cosine similarity: 0.809) and defective DNA mismatch repair (COSMIC Signature 6; cosine similarity: 0.849) (Figure 5G, 5H). C > T and C > G mutations at TpCpN trinucleotides were attributed to the overactivity of the AID/APOBEC family of cytidine deaminases. These data indicate that the overactivity of APOBEC family genes may be involved in HNSCC tumorigenesis and immune outcome.

### Copy number alterations of the TME phenotypes

**Figure 6:**
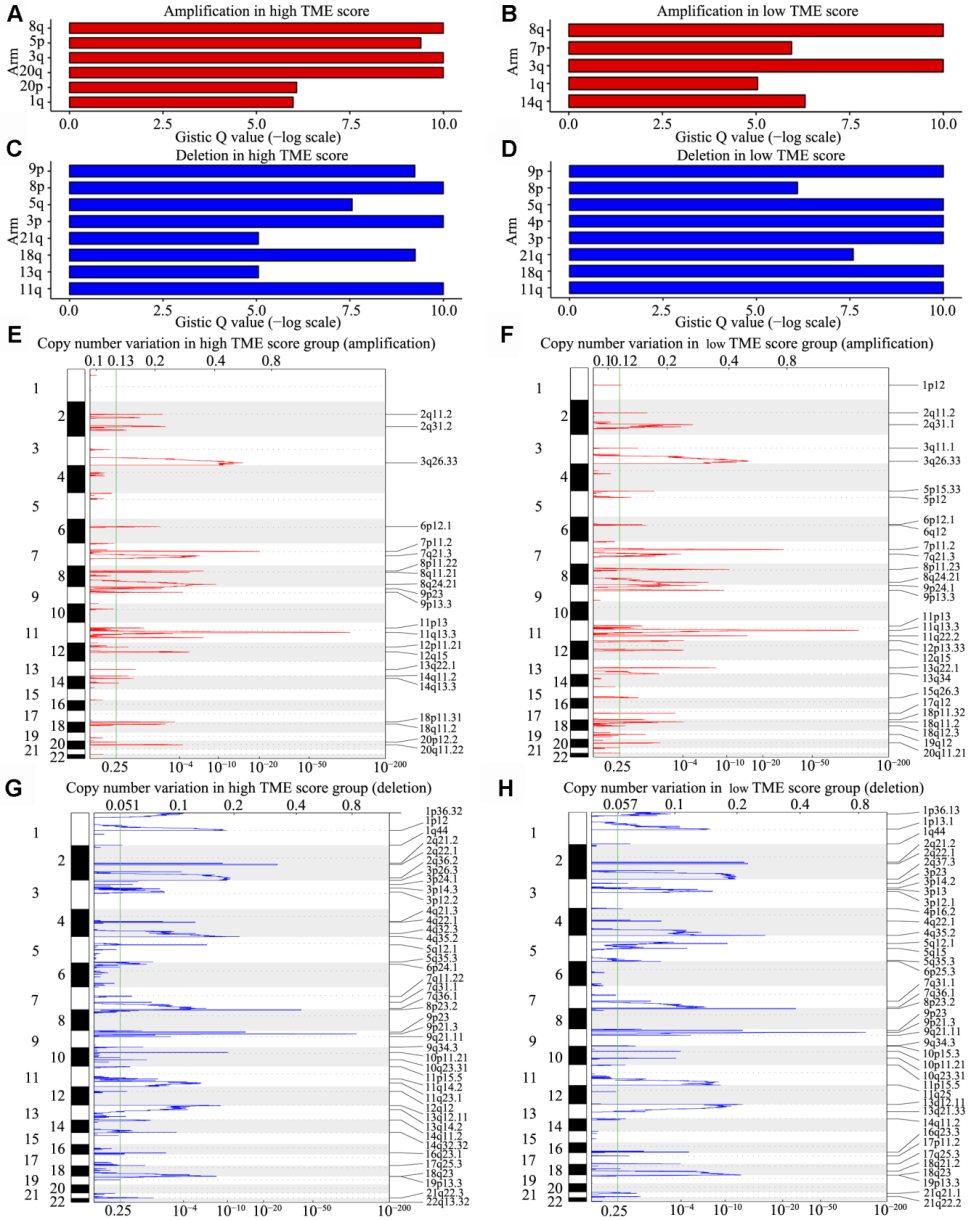
**CNV analysis in HNSCC.** (**A**–**D**) CNV at arm level. The bar graphs show the frequency of arm-level CNV amplification (**A**, **B**) and deletion (**C**, **D**), the vertical axis denotes chromosome arms. (**E**–**H**) CNV at focal regions detected by GISTIC v2·0. Regions of recurrent focal amplifications (**E**, **F**) and focal deletions (**G**, **H**) in the high and low TME score groups are plotted by false discovery rate (x-axis) for each chromosome (y-axis). Dashed lines represent the centromere of each chromosome.

We observed that 98% HNSCC samples had CNVs at the chromosome arm level, including loss at 3p, 4p, 5q, 8p, 9p, 11q, 13q, 18q and 21q and gain at 1q, 3q, 5p, 8q, 14q, 20p and 20q. Analyses using GISTIC indicated that the chromosome arm level had a significant gain at 8q and 20q in the high TME score group (Figure 6A) and at 8q, 3q and 3p in the low TME score group (Figure 6B); moreover, the chromosome arm level had a significant loss at 8p, 3p and 11q in the high TME score group and at 9p, 5q and 4p in the low TME score group (Figure 6C, 6D). Similar SNV and CNV profiles along with similar RNA expression patterns supported that HNSCC belongs to the “squamous” molecular subtype, as identified by Hoadley et al. [29]. We also identified 13 focal regions with recurrent gain of copy number and 20 regions with recurrent loss of copy number (both q < 1e−4), in which many genes have previously been identified to be tumor-associated genes (Supplementary Table 4). Among them, three recurrent focal amplifications (11q13.3, 7p11.2 and 3q26.33) (Figure 6E, 6F) and three recurrent focal deletions (9p21.3, 8p23.2 and 4q35.2) (Figure 6G, 6H) were identified for the first time in the TME score groups.

### Integrated genomic landscape of the TME score phenotype

**Figure 7:**
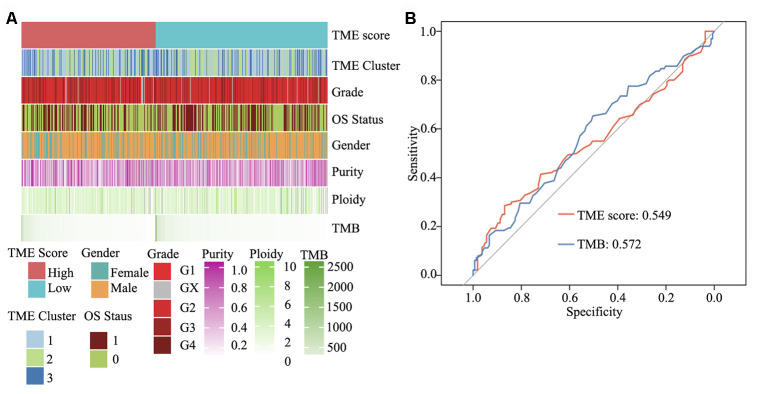
**Clinical and integrated genomic landscape of HNSCC with the TME score phenotype.** (**A**) Comprehensive genomic landscape of HNSCC. (**B**) Prediction immunotherapy effect of TME score by ROC analysis.

To elucidate the molecular characteristics of HNSCC, according to information pertaining to the TME clusters and TME score groups, as well as tumor purity, malignant cell ploidy, tumor mutation burden (TMB) and clinical information (age, grade and OS status), a comprehensive genomic landscape of HNSCC samples was integrated and has been depicted in Supplementary Table 5. As shown in Figure 7A and Supplementary Figure 1C and 1D, no difference was present between the high and low TME score groups with regard to tumor ploidy and malignant cell purity. However, consistent with TMB, TME score was able to significantly differentiate between high- and low-risk HNSCC. TMB, in concert with PD-L1 expression, is reportedly a useful biomarker for immune checkpoint blockade selection in diverse cancers [30]. Herein ROC analysis showed that in comparison with TMB, TME score is a similar efficacious biomarker to determine the effectiveness of immunotherapy in patients with HNSCC (area under ROC: 0.549 vs. 0.572, P = 0.64) (Figure 7B).

## DISCUSSION

HNSCC is a heterogeneous disease of the upper aerodigestive tract, encompassing distinct histological types, different anatomical sites and even HPV+ or EBV+ cancers. Dysfunctional immune cells in patients with recurrent/metastatic HNSCC can be repaired using immunotherapies in combination with conventional treatment methods. In this study, our findings indicated that assessing the immune score via the TME signature provided a potent predictor of survival in patients with HNSCC. Functional analysis of TME signature genes suggested that they are involved in the activation and inhibition of immune responses. Mutational signature and CNV analyses indicated that somatic mutations in tumor DNA or chromosome arm can give rise to neoantigens and mutation-derived antigens, which are recognized and targeted by the immune system, thereby activating the immune response. We report that TME score can act as an immunotherapy biomarker for HNSCC.

Immune checkpoint blockade therapy that impedes the PD-1/PD-L1 and anti-CTLA-4 pathway can increase OS in patients with advanced melanoma, non-small-cell lung cancer, urothelial cancer, renal cell carcinoma and other cancer types [31–35]. However, such a response is observed in only a minority of patients. Several studies have reported that PD-1 and PD-L1 expression, microsatellite instability status and mutation load are not robust biomarkers for predicting the benefits of immune checkpoint blockade [36–38]. Thus, it is of utmost importance to screen effective biomarkers for checkpoint immunotherapy. HNSCC, similar to all other cancers, results from a stepwise accumulation of genomic instability, chromosomal aberrations and genetic mutations [39]. Within cancer tissues, arising mutant cells strive for metabolism, avoid immune surveillance and in collaboration with the extracellular matrix, tumor stroma, immune cells, vessel remodelling and diverse immunoinhibitory soluble or membrane-bound cytokines, establish a unique TME [40]. Tekpli et al. reported an independent poor-prognosis subtype of breast cancer defined by a distinct TME [41]. Zeng et al. also reported that TME characteristics could be used to interpret the response of gastric tumors to immunotherapies [42]. Herein our findings provide clarity on the comprehensive landscape of the interactions between the clinical characteristics of HNSCC and tumor-infiltrating immune cells. With the application of computational algorithms, a methodology was established to determine TME score.

Herein we used CIBERSORT to evaluate differential immune cell infiltration in paired HNSCC and adjacent normal tissues, and the results revealed considerable differences in the immune cell fraction in both the intra- and intergroups. Our results also uncover details pertaining to the infiltration of LM22 immune cell subpopulations in HNSCC, with the proportion of macrophages being >45% (M0 = 27%, M2 = 9% and M1 = 9%). CD4+ memory activated T cells, resting NK cells, regulatory T cells, CD8+ T cells, follicular T cells, monocytes, resting mast cells, plasma cells, naïve B cells and memory B cells were risk factors for OS, while neutrophils, activated mast cells, activated NK cells, resting memory CD4+ T cells, naïve CD4+ T cells, M0 macrophages, M2 macrophages and eosinophils were favourable factors for OS. HNSCC samples were clustered into three main clusters: low, intermediate and high immune infiltration. Patients in the high immune infiltration cluster showed the best survival probability than those in the other two clusters.

Integrated analyses revealed that TME score is a prognostic biomarker for HNSCC; in particular, naïve B cells, regulatory T cells and follicular T cells in the high TME score group were associated with an improved outcome, whereas neutrophils and activated mast cells in the low TME score group were associated with poorer outcome. Further, the involvement of several immune activation pathways suggested that TME score is a predictive biomarker to further advance precision immunotherapy of HNSCC. Higher non-synonymous mutational burden has been associated with an improved overall response rate, durable clinical benefits and progression-free survival in patients treated with ICIs [43]. C > T and/or C > G mutations at TpCpN trinucleotides were attributed to the overactivity of the AID/APOBEC family of cytidine deaminases. Although the APOBEC family of proteins might serve as endogenous carcinogenic mutagens [44], they also play crucial roles in the innate immune response to viral infections by modifying the viral genome [45, 46]. Through ROC analysis, we found that TME score showed a predictive value similar to that of TMB, which indicates that it may act as an independent biomarker for immunotherapy.

To summarize, our results provide a comprehensive view of the cellular, molecular and genetic factors associated with TME infiltration patterns and define a potential mechanism by which tumors respond to immunotherapy. We believe that TME score is a useful biomarker that can effectively predict immunotherapy outcome in patients with HNSCC.

## MATERIALS AND METHODS

### Data acquisition and processing

RNA-seq data (N = 546) and survival data (N = 530) were obtained from TCGA database (https://portal.gdc.cancer.gov/projects/TCGA-HNSC). Patients diagnosed with HNSCC and with clinicopathological and survival information (N = 500) were transformed as original read counts. Next, genes with low expression levels were removed using the filterByExpr function of edgeR. The expression data were then transformed using voom to facilitate the evaluation of immune cell subpopulations by CIBERSORT.

### Evaluation of tumor-infiltrating immune cells

To evaluate the number of each type of tumor-infiltrating immune cells, we applied the original CIBERSORT gene signature file LM22, which defines 22 immune cell subpopulations, to analyse datasets pertaining to HNSCC. Gene expression datasets were prepared using standard annotation files and data uploaded to the CIBERSORT web portal (http://cibersort.stanford.edu/), with the algorithm run using the default signature matrix at 1,000 permutations [20].

### Consensus clustering of TME-infiltrating patterns

Different TME cell infiltration patterns were grouped using hierarchical agglomerative clustering based on Euclidean distance and Ward’s linkage. In addition, we used an unsupervised clustering method (K-means) for dataset analysis to identify TME cell infiltration patterns and to classify patients for further analyses [47]. The consensus clustering algorithm was used to detect the number of clusters in the meta-dataset. This was performed using the *ConsensusClusterPlus* R package and was repeated 1,000 times to ensure the stability of classification [48].

### DEGs associated with the TME phenotypes

To identify genes associated with TME cell infiltration patterns in patients with HNSCC, we grouped the TME cluster into three classes: TME cluster 1, 2 and 3. DEGs among these three clusters were determined using the R package limma, which implements an empirical Bayesian approach to estimate changes in gene expression levels using moderated *t*-tests. DEGs among the TME clusters were determined by significance criteria (P < 1e−3 and |log2FC| > 1), as implemented in the R package limma.

### TME gene signature analyses

The construction of TME metagenes was performed as follows. Each DEG among TME cluster 1, 2 and 3 was standardized across all samples. An unsupervised clustering method (K-means) was used for DEG analyses to classify patients into three groups for further analyses. The random forest classification algorithm was used for dimension reduction to reduce noise or redundant class-specific DEGs in TME clusters. DEGs among the TME clusters were annotated using the clusterProfiler R package [49]. A consensus clustering algorithm was applied to define the cluster of genes and to calculate the signature score. For gene expression (normalized by RMA or TPM methods) analysis, the expression of each gene in a signature was first transformed into a z-score, and then, principal component analysis (PCA) was performed using the consensus clustering algorithm. Principal component 1 was extracted to serve as the gene signature score. After obtaining the prognostic value of each gene signature score, we used the gene expression grade index method to obtain the TME score of each patient [50]:

TME score = ∑voom(X) – ∑voom(Y)

wherein X represents the signature score of expression value of positive gene clusters, and Y represents the expression level of gene clusters. Patients with HNSCC were therefore assigned to groups based on high or low TME scores using the cut-off value obtained with the maxstat R package; the clinical outcomes were further analysed.

### Functional and pathway enrichment analysis

Gene ontology terms (cellular component, biological process and molecular function) of TME gene signatures were identified using the clusterProfiler R package [49]. Gene set enrichment analysis of DEGs with high or low TME scores was performed based on the MSigDB database (Broad Institute) [51]. Broad hallmarks and specific pathways of interest from curated gene sets/canonical pathway collection were all included.

### Mutational signature analysis

Mutational signature was first identified using the BayesNMF algorithm. The count of somatic mutations was calculated for each type of substitution (96 trinucleotide mutation contexts) to generate a mutational catalogue. We then ran the Bayesian NMF 1,000 times with the hyperparameter for the inverse gamma prior setting to 10 (*a* = 10); the iterations were terminated when the tolerance for convergence was < 10 e–7, and half-normal was selected as “pirors” for this algorithm [52]. Signatures identified following matrix factorization were scaled and the results were compared to the Catalogue of Somatic Mutations in Cancer (COSMIC) signature database. A cosine similarity value was then estimated for the best possible match [53]. For signature enrichment analysis, we used matrix H, containing signature exposures for every sample in every signature. Using K-means clustering, the samples were grouped into r clusters, thereby assigning samples to an identified signature.

For apolipoprotein B-editing catalytic polypeptide-like subunit (APOBEC)-based enrichment analysis, we used the method described by Roberts et al. [44] to estimate an enrichment score, which defined the strength of APOBEC-related mutagenic processes for every tumor sample in Mutation Annotation Format. Briefly, the enrichment of C > T mutations occurring within over all C > T mutations in a given sample was compared to background cytosines and occurring around ±20 bp of mutated bases. We further used this method to identify genes associated with APOBEC enrichment by classifying samples as APOBEC-enriched (enrichment score > 2) and non-APOBEC-enriched (enrichment score < 2), followed by using one-way Fisher’s exact tests to identify genes overrepresented among APOBEC-enriched samples.

### Analysing CNVs

To identify variant regions that drive cancer pathogenesis, the Genomic Identification of Significant Targets in Cancer (GISTIC) algorithm was used to detect genomic regions manifesting amplifications (copy number > 1) or deletions (copy number < −1) [54]. G-score was determined to evaluate the amplitude of aberrations and frequency of occurrence in variant regions. Briefly, GISTIC v2.0 was used to define the prepared CNV profiles for all genes in the 488 HNSCC samples. False discovery rate q-values were assigned to each variant region. “Peak regions,” also known as significantly aberrant regions, indicated the greatest frequency and amplitude of aberrations [55, 56].

### Analysing somatic DNA copy number alterations

To elucidate tumor purity and malignant cell ploidy from the somatic DNA copy number alterations, ABSOLUTE was used to detect subclonal heterogeneity and somatic homozygosity and to calculate statistical sensitivity to identify specific aberrations. Briefly, as described by Carter et al. [57], based on the CNV data of somatic DNA copy number alterations, pre-designed cancer karyotypes and somatic mutation frequencies were scored and integrated. The highest score was considered to represent the optimal model, and tumor purity and malignant cell ploidy were then detected using the ABSOLUTE R package limma.

### Statistical analysis

Student’s *t*-test and ANOVA were utilized to compare continuous and discrete variables, respectively. Pearson’s chi-squared test was used for comparing categorical clinicopathological variables, and survival probability and differences were analysed using log-rank test and the Cox proportional hazards model (multivariate analysis). Statistical analyses were performed using standard R packages (version 3.5.2). P < 0.05 indicated statistical significance.

## Supplementary Material

Supplementary Figures

Supplementary Table 1

Supplementary Table 2

Supplementary Table 3

Supplementary Table 4

Supplementary Table 5
