# Supplementary material for: Comprehensive characterization of the tumor microenvironment for assessing immunotherapy outcome in patients with head and neck squamous cell carcinoma
Source: Aging (Albany NY). 2020 Nov 18;12(22):22509–26. doi: 10.18632/aging.103460 (PMC7746351; doi:10.18632/aging.103460)
Supplement: Supplementary Figures [file aging-12-103460-s001..pdf]

SUPPLEMENTARY FIGURE

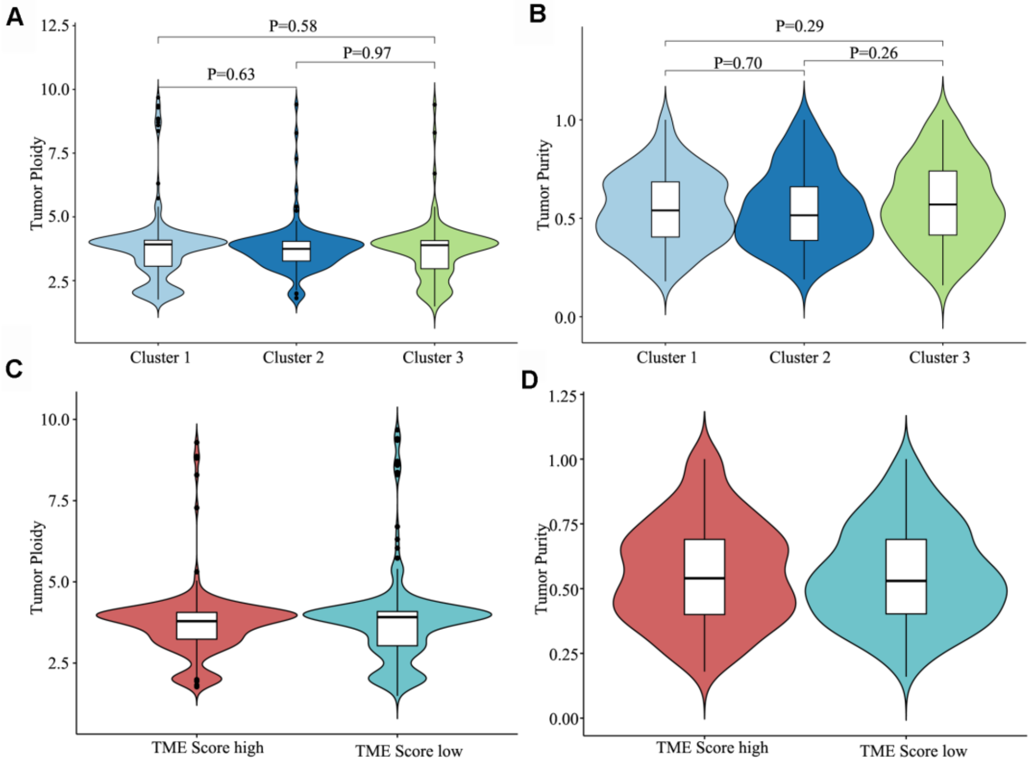

Supplementary Figure 1. Tumor ploidy and malignant cell purity in HNSCC samples. Ploidy (**A**) and purity (**B**) in TME cluster-1, -2 and -3. Ploidy (**C**) and purity (**D**) in the high and low TME score groups.
